# Limiting Postpartum Weight Retention in Culturally and Linguistically Diverse Women: Secondary Analysis of the HeLP-her Randomized Controlled Trial

**DOI:** 10.3390/nu14142988

**Published:** 2022-07-21

**Authors:** Mingling Chen, Siew Lim, Cheryce L. Harrison

**Affiliations:** 1 Monash Centre for Health Research and Implementation, School of Public Health and Preventive Medicine, Monash University, Clayton, VIC 3168, Australia; mingling.chen@monash.edu; 2 Eastern Health Clinical School, Monash University, Box Hill, VIC 3128, Australia; siew.lim1@monash.edu; 3 Diabetes and Endocrine Unit, Monash Health, Clayton, VIC 3168, Australia

**Keywords:** ethnicity, lifestyle intervention, postpartum weight retention, pregnancy

## Abstract

Postpartum weight retention (PPWR) contributes to maternal obesity development and is more pronounced in culturally and linguistically diverse (CALD) women. Our antenatal healthy lifestyle intervention (HeLP-her) demonstrated efficacy in reducing PPWR in non-Australian-born CALD women compared with Australian-born women. In this secondary analysis, we aimed to examine differences in the intervention effect on behavioral and psychosocial outcomes between Australian-born and non-Australian-born women and explore factors associated with the differential intervention effect on PPWR. Pregnant women at risk of gestational diabetes (Australian-born *n* = 86, non-Australian-born *n* = 142) were randomized to intervention (four lifestyle sessions) or control (standard antenatal care). PPWR was defined as the difference in measured weight between 6 weeks postpartum and baseline (12–15 weeks gestation). Behavioral (self-weighing, physical activity (pedometer), diet (fat-related dietary habits questionnaire), self-perceived behavior changes), and psychosocial (weight control confidence, exercise self-efficacy, eating self-efficacy) outcomes were examined by country of birth. Multivariable linear regression analysis was conducted to assess factors associated with PPWR. The intervention significantly increased self-weighing, eating self-efficacy, and self-perceived changes to diet and physical activity at 6 weeks postpartum in non-Australian-born women, compared with no significant changes observed among Australian-born women. Intervention allocation and decreased intake of snack foods were predictors of lower PPWR in non-Australian-born women. Results indicate that the HeLP-her intervention improved dietary behaviors, contributing to the reduction of PPWR in CALD women. Future translations could prioritize targeting diet while developing more effective strategies to increase exercise engagement during pregnancy in this population.

## 1. Introduction

Overweight and obesity are significant global health challenges, affecting approximately 40% of women worldwide [1]. Pregnancy is recognized as a high-risk period for accelerated weight gain in women. Excessive gestational weight gain (GWG) contributes to postpartum weight retention (PPWR) and increases the risk of adverse outcomes in subsequent pregnancies, including preeclampsia, gestational diabetes mellitus (GDM), cesarean delivery, and large-for-gestational-age birth, as well as the development of long-term maternal obesity, cardiovascular disease, and metabolic syndrome in later life [2,3]. It is documented that PPWR is more pronounced among underserved populations, including women from culturally and linguistically diverse (CALD) backgrounds [4]. Observational studies in the US and Europe have shown women from South Asian, Middle Eastern, and African groups experience more weight retention postpartum than women of European background [5,6,7]. Contributory factors include lower socioeconomic status, higher psychosocial stress, and suboptimal lifestyle behaviors such as greater energy intake and less physical activity during and after pregnancy [5,6,8].

Lifestyle intervention in pregnancy comprising a healthy diet and/or physical activity optimizes GWG [9], thus promoting a return to pre-pregnancy weight after childbirth. Despite accumulated evidence on the benefits of such interventions during and following pregnancy, few studies have explored variation in intervention efficacy by ethnicity [10,11]. Given the increased risk of retaining weight postpartum in CALD women [4], understanding intervention effects on postpartum outcomes and associated factors that contribute to intervention effects in CALD groups is important to identify facilitating factors and effective strategies for weight management during pregnancy for these high-risk populations.

The Healthy Lifestyle in Pregnancy (HeLP-her) study is a low-intensity lifestyle intervention previously conducted in Australian antenatal care settings to optimize GWG and PPWR in women at increased risk of GDM [12,13]. The study population was ethnically diverse, with ~65% of women non-Australian-born. We have previously demonstrated the efficacy of the intervention, which was more effective in reducing weight retention at 6 weeks postpartum in non-Australian-born women (intervention 1.13 ± 4.11 kg vs. control 3.66 ± 5.47 kg, *p* < 0.01) compared with Australian-born women (−0.56 ± 4.93 kg vs. −0.74 ± 5.14 kg, *p* = 0.87) [13], yet exploratory analysis was not undertaken to elucidate this differential intervention effect. Here, this secondary analysis aims to: (1) examine differences in behavioral and psychosocial outcomes at 6 weeks postpartum between Australian-born and non-Australian-born women to further our understanding of ethnic variability in response to the intervention and (2) explore factors associated with the differential intervention effect on postpartum weight retention to provide insight into future intervention design and translation to benefit higher risk, CALD populations.

## 2. Materials and Methods

### 2.1. Study Design

Detailed study design and methods have been previously described [12,13]. In brief, women were recruited at three large metropolitan tertiary teaching hospitals in Victoria, Australia, between June 2008 and October 2010, combining over 8600 births per year. The hospitals serve a region of ethnically and culturally diverse populations with over one-third of residents non-Australian-born, comprising the largest refugee and migrant community in Victoria [14]. According to the Australian Bureau of Statistics, the region is classified of “average socioeconomic advantage” [15]. Inclusion criteria were: gestational age ≤ 15 weeks, singleton pregnancy, body mass index (BMI) ≥ 25 kg/m^2^ (or BMI ≥ 23 kg/m^2^ if high-risk ethnicity, i.e., Asian, African, and Polynesian [16]), and at increased risk of GDM as identified by a validated risk prediction tool [12,13,17]. Exclusion criteria included multiple pregnancies, BMI ≥ 45 kg/m^2^, type 1 or 2 diabetes diagnosis, pre-existing chronic medical conditions, and non-English speaking women. Eligible women were randomized to intervention or control through computer-generated randomized sequencing. All women received standard antenatal care. Informed consent was obtained from all participants. The study was approved by the Southern Health Research Advisory and Ethics Committee. The trial was registered on the Australian New Zealand Clinical Trial Registry (ACTRN12608000233325) [12,13].

### 2.2. Intervention Group

Underpinned by the Social Cognitive Theory, the low-intensity behavior change program included four, 45 min individual lifestyle sessions delivered at 14–16, 20, 24, and 28 weeks gestation by a trained health coach [12,13]. The sessions delivered simple, pregnancy-specific healthy eating and physical activity messages based on National guidelines [18,19], as well as healthy GWG information according to the National Academy of Medicine (previously, Institute of Medicine [IOM]) guidelines [20]. Behavior change strategies were utilized to practice and increase self-management, including personal goal setting, problem-solving, action planning, self-monitoring, addressing barriers, and relapse prevention [12,13]. Ongoing support with mobile phone SMS messages was provided throughout the intervention. In addition, two healthy lifestyle postcards were distributed at 30 and 34 weeks gestation to reinforce behavior change and maintain engagement [12,13].

### 2.3. Control Group

The control group received one individual 15 min education session based on the population-based Australian Dietary and Physical Activity Guidelines [18,19] at baseline, as well as written pamphlets. No further support was provided during the study period [12,13].

### 2.4. Data Sources

Data collected at baseline (12–15 weeks gestation) and 6 weeks postpartum was used for this secondary analysis.

#### 2.4.1. Demographics

Demographic information including age, country of birth, years lived in Australia, education, employment, household income, parity, and breastfeeding status, were collected using a structured self-administered questionnaire. Women were grouped by country of birth into two categories: Australian-born (born in Australia) and non-Australian-born (born outside of Australia). Those non-Australian-born are referred to as CALD women [21,22].

#### 2.4.2. Anthropometrics

Anthropometric measurements (height, weight) were conducted by a registered nurse who was blinded to group allocation. Weight was measured to the nearest 0.1 kg on an electronic scale (Tanita model BWB-800 Digital Scale, Wedderburn Scales, Melbourne, Australia). The primary outcome was PPWR, calculated as the difference in measured weight (kg) between baseline and 6 weeks postpartum.

#### 2.4.3. Health Behaviors

##### Physical Activity

The Yamax Digiwalker SW-700 Pedometer (Yamax Corporation, Tokyo, Japan) was used to assess the free-living step count per day as a tool with demonstrated accuracy in pregnancy, as previously reported [23]. Participants were asked to wear a sealed pedometer for a minimum of three to seven consecutive days during waking hours. Readings were processed to provide the average daily step count according to the total days worn.

##### Diet

Dietary behaviors related to adopting low-fat diets were assessed using a 20-item scale that was derived from the Fat-Related Dietary Habits Questionnaire [24]. The 20-item scale included five dimensions: “avoid fat as flavoring”, “modify meats”, “avoid frying”, “substitute lower-fat products”, and “replace foods with fruits and vegetables”. Responses to the items were on a 3-point scale (“usually”, “sometimes”, and “rarely or never”) and were coded through 1 to 3 to positively correlate with fat intake. An additional “don’t eat this food” option was also provided for each item, coded as missing data. A summary mean score was calculated.

##### Self-Weighing

Self-reported frequency of self-weighing was collected via questionnaire. Data were dichotomized as frequent (“daily”, “weekly” or “monthly”) or not frequent (“occasionally” or “never”) self-weighing.

#### 2.4.4. Psychosocial Measures

##### Risk Perception

Risk perception for excess GWG and development of GDM was assessed on a 4-point scale adapted from the theory of health stage of change [25]. Data were dichotomized as no perceived risk (“definitely no risk” or “not really at risk”) or perceived risk (“slight risk” or “high risk”).

##### Weight Control Confidence

Confidence for weight control was assessed by asking “how confident are you that you can control your weight gain if you wished” and “how confident are you that you can control your weight gain if you experienced difficulties”, which were adapted from the Chronic Disease Self-Efficacy Scale [26]. Responses were on a 10-point scale from “not at all confident” (1) to “totally confident” (10). A summary mean score was calculated, with higher scores indicating higher confidence.

##### Exercise and Eating Self-Efficacy

Self-efficacy for exercise and eating behaviors was assessed using a 14-item scale derived from a validated scale developed by Sallis et al. [27]. The 14 items consisted of four subscales: “sticking to it (exercise)”, “making time for exercise”, “sticking to it (eating)”, and “reducing calories”. Responses were on a 5-point scale from “not at all confident” (1) to “extremely confident” (5). The mean score of each subscale was calculated, with higher scores indicating higher self-efficacy.

#### 2.4.5. Other Measures

At 6 weeks postpartum, participants were asked to report their self-perceived change in physical activity and diet since participating in the program on a 4-point scale; responses were dichotomized as perceived change (“lots of changes”, “some changes”, or “minor changes”) or no perceived change (“no changes at all”). If a perceived change in physical activity or diet was reported, specific changes in these behaviors were collected. Participants were also asked to evaluate the program on assisting them in optimizing physical activity and healthy diet on a 4-point scale; responses were dichotomized as positive (“very helpful”, “helpful”, or “slightly helpful”) or negative evaluation (“not helpful at all”). The number of sessions attended by participants was also recorded to assess the intervention compliance.

### 2.5. Statistical Analysis

As our primary analysis demonstrated a differential intervention effect on PPWR by country of birth (i.e., Australian-born vs. non-Australian-born) [13], here our secondary analysis was conducted by stratifying data according to country of birth to explore the differential responses in behavioral and psychosocial outcomes and factors associated with the effect of country of birth on PPWR based on the primary finding. Data are presented as mean ± standard deviation (continuous variables) and proportions (categorical variables). Differences in baseline or categorical outcome measures between groups (Australian-born vs. non-Australian-born, intervention vs. control) were compared using independent sample *t* tests for continuous variables and chi-square tests or Fisher’s exact tests for categorical variables. Least squares means and their differences between groups (intervention vs. control) derived from multivariable linear regressions that adjusted for baseline variables (age, education, work, parity, BMI, self-weighing, risk perception of excess GWG, risk perception of GDM, eating self-efficacy) were used to present changes in continuous outcome measures. Due to the differing proportions of baseline BMI status (i.e., overweight or obesity) between Australian-born and non-Australian-born participants, we examined the potential interaction effect between intervention, baseline BMI status, and country of birth on the primary outcome of PPWR by conducting a three-way interaction term in linear regression analysis, with the intervention effect on PPWR stratified according to baseline BMI (i.e., Australian-born with overweight, Australian-born with obesity, non-Australian-born with overweight, non-Australian-born with obesity). To assess factors associated with PPWR, a multivariable linear regression model was constructed by including demographic, anthropometric, behavioral, and psychosocial variables with *p* < 0.1 on univariate analyses and using backward elimination to remove variables at *p* > 0.05. The model was adjusted for intervention allocation, age, baseline BMI and parity, the latter three as clinically relevant variables associated with PPWR. We used complete case analysis (i.e., all available data included in the analysis) as data were deemed missing at random, negating the need for multiple imputations [12,13]. Statistical significance was set at an α level of *p* < 0.05. All analyses were performed with SAS 9.4 (SAS Institute Inc., Cary, NC, USA).

## 3. Results

The HeLP-her study randomized 228 eligible women, with 86 Australian-born and 142 non-Australian-born. Over half (57%) of the non-Australian-born women were from South Asia (primarily India, Sri Lanka, Bangladesh, and Pakistan), 23% from East and Southeast Asia (primarily China, Vietnam, Malaysia, and Indonesia), and 12% from the Middle East, Africa, and Pacific Islands. Most (80%) of the non-Australian-born women had resided in Australia for less than 10 years. At 6 weeks postpartum, 202 women were followed up and completed primary outcome weight measurements (76 Australian-born and 126 non-Australian-born), with an overall attrition rate of 11.4% attributed to miscarriage or stillbirth, pre-term birth, and loss to contact (Figure S1).

### 3.1. Baseline Characteristics

Table 1 shows the baseline characteristics of the participants. Compared to Australian-born women, non-Australian-born women were younger, had a higher education level, were less likely to be employed, and were more likely to be primiparous. The mean BMI of non-Australian-born women was 28.0 ± 4.4 kg/m^2^, lower than 34.3 ± 5.5 kg/m^2^ in Australian-born women, with 22.5% and 73.3% being obese in the two groups, respectively. In addition, non-Australian-born women were less likely to weigh themselves frequently and had a lower risk perception for excess GWG and GDM at baseline than Australian-born women. Conversely, eating self-efficacy was higher in non-Australian-born compared with Australian-born women. There were no significant differences in the baseline levels of physical activity, fat-related dietary behaviors, weight control confidence, and exercise self-efficacy between the two groups.

### 3.2. Intervention Effect

The intervention effects on behavioral and psychosocial measures are shown in Table 2 and Table 3. Non-Australian-born women in the intervention group had significantly improved eating self-efficacy in “reducing calories” (intervention effect 0.68 (95%CI 0.15, 1.21), *p* = 0.013), and were more likely to report they had made changes to physical activity (intervention 89.4% vs. control 65.9%, *p* = 0.008) and diet (97.9% vs. 73.2%, *p* < 0.001) at 6 weeks postpartum, compared to the control group. A higher proportion of non-Australian-born women in the intervention group reported specific changes to diet than those in the control group, including increased fruit and vegetable consumption, increased low-fat dairy products, decreased intake of snack foods, and decreased takeaway and convenience foods (all *p* < 0.05). Frequent self-weighing in non-Australian-born women increased from 48.3% at baseline to 72.3% at 6 weeks postpartum (*p* = 0.013) in the intervention group, with no significant change from baseline (31.9%) to 6 weeks postpartum (36.6%) in the control group (*p* = 0.645). No significant differences between intervention and control were found in changes in physical activity, fat-related dietary behaviors, weight control confidence, and exercise self-efficacy in non-Australian-born women (all *p* > 0.05). In Australian-born women, none of the measures significantly differed between intervention and control. Frequent self-weighing in Australian-born women was similar over time (baseline to 6 weeks postpartum) irrespective of intervention allocation.

Most Australian-born (96.4%) and non-Australian-born women (95.4%) had a positive evaluation of the program at 6 weeks postpartum. Of the women allocated to the intervention, 94.9% Australian-born and 90.8% non-Australian-born women attended all four sessions, with no significant differences in the intervention compliance (*p* = 0.707).

### 3.3. Interaction Analysis

Table S1 shows the intervention effect according to country of birth and baseline BMI. There was a significant intervention effect on reducing PPWR in non-Australian-born women with overweight (intervention 1.97 ± 3.92 kg vs. control 3.98 ± 5.53 kg, *p* = 0.040), as well as a trend toward less PPWR in non-Australian-born women with obesity (−1.42 ± 3.77 kg vs. 2.03 ± 5.03 kg, *p* = 0.052). In contrast, no significant intervention effect was revealed among Australian-born women regardless of BMI status (*p* > 0.05). No interaction effect was found between intervention, baseline BMI status, and country of birth (*p* = 0.237).

### 3.4. Regression Analysis

The regression results are presented in Tables S2 and S3. The multivariable analysis showed intervention allocation and decreased intake of snack foods were independent predictors of lower PPWR in non-Australian-born women. Other demographic, anthropometric, behavioral, and psychosocial factors did not significantly impact their PPWR. In Australian-born women, increased time spent on physical activity sessions was the only factor significantly associated with PPWR.

## 4. Discussion

We previously reported a greater effect of the HeLP-her intervention on reducing weight retention at 6 weeks postpartum in non-Australian-born women compared with Australian-born women [13]. In this study, we expand on these findings by examining factors that may be related to the differential intervention effect, including demographic and anthropometric characteristics as well as behavioral and psychosocial factors targeted by the intervention. Here, we found that non-Australian-born women receiving the intervention had improved self-efficacy for dietary behaviors, were more likely to self-weigh frequently, and reported changes to diet and physical activity at 6 weeks postpartum, compared to standard antenatal care. In contrast, we did not observe any significant changes in behavioral or psychosocial measures among Australian-born women following the intervention. Exploratory analysis showed intervention allocation and decreased intake of snack foods were predictors of lower weight retention postpartum in non-Australian-born women.

The HeLP-her intervention is non-prescriptive, utilizing simple messages on healthy eating and physical activity aligned with national dietary and physical activity recommendations, underpinned by practicing skills in self-management. Weight self-monitoring has been identified as an essential component in weight management, enabling immediate adjustment to weight-related behaviors as well as reinforcement [28]. Yet previous studies in the US have shown ethnic minority groups are less likely to report frequent self-weighing compared to their white counterparts [29,30], as consistent with the baseline findings in non-Australian-born women in the HeLP-her study. This could be partly attributable to decreased risk perception related to weight gain during pregnancy and lower awareness of GDM risk, as observed here at baseline. Encouragingly, our results showed the HeLP-her intervention significantly improved the self-weighing behaviors in non-Australian-born women, despite no improvement observed in Australian-born women. The greater improvement in self-weighing frequency following the intervention among non-Australian-born women suggests receptiveness to the intervention messaging with amenability to behavior change in this group.

The intervention focused on small, sustainable behavior adjustments using individualized goal setting and action planning. Therefore, it is not surprising that we were unable to detect significant changes in physical activity measured by daily step counts or fat-related dietary behaviors measured in dietary scores in non-Australian-born women, per our previous findings [12,13]. It is possible that the measurement tools used were less sensitive to detect the small behavioral changes encouraged as part of the intervention. Despite no differences in quantitatively measured physical activity or dietary behaviors, non-Australian-born women were more likely to report perceived changes in diet and physical activity. Particularly, changes in several specific dietary behaviors were reported, among which decreased intake of snack foods was found to be associated with less PPWR on the multivariable analysis. In line with this, non-Australian-born women had higher eating self-efficacy in “sticking to it” at baseline, which persisted into 6 weeks postpartum. Furthermore, they had greater improvement in self-efficacy of “reducing calories” after the intervention. These are reflective of improved confidence in the ability to make behavior changes as well as increased commitment and practicing behavior change towards dietary behaviors. In contrast to the positive changes seen in diet, we did not find any changes in specific physical activity behaviors related to the increasing number, time, or intensity of exercise, nor the improvement in exercise self-efficacy over time among non-Australian-born women. Previous studies have found a lower level of physical activity during pregnancy among Asians, Middle Easterners, and Africans compared to white populations [31,32,33]. It is shown that women’s beliefs, attitudes, barriers, and intentions towards exercise during pregnancy differ between cultures [32]. For women from CALD backgrounds, safety concerns about exercising during pregnancy is a significant barrier, reflected by cultural beliefs [32,34]. For example, in traditional Chinese culture, pregnant women are advised to restrict exercise due to concern of miscarriage [35]. For this reason, it is plausible that dietary behaviors may be more readily modified, with fewer barriers to behavior change, than physical activity behaviors in CALD women during pregnancy. However, given physical activity is safe and associated with optimized weight and reduction in complications during pregnancy [36,37], it is imperative to find ways to address barriers and improve health knowledge towards physical activity in this population.

In contrast to non-Australian-born women, we did not find significant intervention effects on behavioral, psychosocial, and weight outcomes among Australian-born women irrespective of BMI status (i.e., overweight or obesity). This is consistent with previous Australian-based trials, including the large LIMIT randomized trial conducted in predominantly white women with overweight or obesity, which reported no differences in GWG between antenatal care and lifestyle advice following six intervention sessions throughout pregnancy [38]. In our study, women born in Australia had a higher baseline risk perception, potentially reflecting a higher level of health literacy and confidence or familiarity in access to healthcare services and information [39]. Therefore, the low-intensity intervention format utilizing simple health messaging may not have been sufficient to influence further behavior change towards diet, physical activity and self-management behaviors. Future research, potentially including more tailored or prescriptive intervention, is needed to evaluate outcomes with different intervention types and intensities in this population.

### Strengths and Limitations

Here, we used a rigorous study design, utilized robust measures (including a validated GDM screening tool, objective measurement of weight, and pedometer for assessing physical activity), and reported high compliance and high fidelity to the intervention delivery [12,13]. Limitations include the absence of measured dietary intake (e.g., energy intake) and health literacy that may have elucidated findings further [40]. Also, despite a relatively high retention rate (88.6%) at follow-up of 6 weeks postpartum, up to 30% of questionnaire data were missing. Furthermore, the non-Australian-born participants in our study were of moderate socioeconomic advantage and predominantly Asian, reflective of the broader Australian demographic data on migrant populations [41]. As a secondary analysis, our results may not be fully generalizable to all populations, which remained to be confirmed in larger, population-based studies.

## 5. Conclusions

Women from CALD backgrounds experience greater health inequity with an increased risk of adverse health outcomes during pregnancy [42]. Strategies that are accessible, relevant, culturally acceptable, and cost-effective are needed to support health improvement for these women during pregnancy. Our results suggest a low-intensity intervention based on simple health messages alongside routine antenatal care is acceptable and relevant to diverse cultures with demonstrated efficacy in reducing postpartum weight retention among high-risk CALD groups. The improvement in weight outcome appears to derive from small individually driven changes to dietary behaviors during pregnancy, potentially reflective of increased receptiveness to dietary, compared with physical activity, behavior change. Further research is needed to address barriers to exercise in this population to maximize exercise engagement during pregnancy and promote broader health benefits.

## Figures and Tables

**Table 1:** Baseline characteristics of participants.

Variables	Australian-Born (*n* = 86)	Non-Australian-Born (*n* = 142)	*p* Value
Demographics			
Age (years)	32.8 ± 4.4	31.5 ± 4.5	0.037
Education (%)			<0.001
High school or below	29.6	12.1	
Certificate/diploma	40.8	23.5	
Bachelor’s degree or higher	29.6	64.4	
Work (%)			0.004
Full-time	23.5	28.0	
Part-time	43.2	22.0	
No paid work	33.3	50.0	
Household income (%)			0.050
<$40,000	22.2	36.2	
$40,000–80,000	35.8	33.1	
>$80,000	28.4	15.0	
Parity (%)			0.032
Primiparous	32.1	47.0	
Anthropometrics			
Weight (kg)	90.8 ± 16.8	70.5 ± 13.3	<0.001
BMI (kg/m^2^)	34.3 ± 5.5	28.0 ± 4.4	<0.001
BMI (kg/m^2^) (%)			<0.001
Overweight (≤29.9)	26.7	77.5	
Obesity (≥30.0)	73.3	22.5	
Behavioral			
Physical activity (steps/day)	6201.0 ± 2921.4	5570.9 ± 3188.8	0.176
Fat-related dietary behaviors ^a^	1.9 ± 0.3	1.9 ± 0.3	0.694
Frequent self-weighing (%)	62.9	40.9	0.006
Psychosocial			
Perceived risk of excess GWG (%)	85.7	71.4	0.036
Perceived risk of GDM (%)	63.5	40.0	0.004
Weight control confidence ^b^	5.7 ± 2.0	5.9 ± 2.1	0.630
Exercise self-efficacy ^c^			
Sticking to it	2.5 ± 0.9	2.4 ± 0.8	0.500
Making time for exercise	2.3 ± 0.9	2.3 ± 0.8	0.966
Eating self-efficacy ^c^			
Sticking to it	2.6 ± 0.8	3.0 ± 0.9	0.015
Reducing calories	3.4 ± 0.9	3.6 ± 0.7	0.185

BMI, body mass index; GWG, gestational weight gain; GDM, gestational diabetes mellitus. Data are presented as mean ± SD or percentage. ^a^ 1 = usually choose low-fat; 3 = rarely or never choose low-fat. ^b^ 1 = not at all confident; 10 = totally confident. ^c^ 1 = not at all confident; 5 = extremely confident.

**Table 2:** Changes in behavioral and psychosocial measures from baseline to 6 weeks postpartum.

Variables	Australian-Born (*n* = 76)				Non-Australian-Born (*n* = 126)			
	Intervention (*n* = 39)	Control (*n* = 37)	Intervention Effect	*p* Value	Intervention (*n* = 65)	Control (*n* = 61)	Intervention Effect	*p* Value
Behavioral change in								
Physical activity (steps/day)	5135.0 (−14,637.0, 24,907.0)	243.4 (−14,874.5, 15,361.4)	4891.6 (−11,427.0, 21,210.1)	0.537	912.1 (−1473.8, 3298.1)	−1711.2 (−4683.5, 1261.1)	2623.3 (−670.7, 5917.3)	0.114
Fat-related dietary behaviors ^a^	0.07 (−0.19, 0.32)	0.11 (−0.11, 0.33)	−0.04 (−0.25, 0.17)	0.695	−0.07 (−0.24, 0.10)	−0.02 (−0.22, 0.19)	−0.05 (−0.28, 0.17)	0.646
Psychosocial change in								
Weight control confidence ^b^	−0.61 (−3.21, 2.00)	−0.52 (−2.74, 1.70)	−0.09 (−2.24, 2.07)	0.934	0.30 (−0.87, 1.47)	0.55 (−0.88, 1.98)	−0.25 (−1.82, 1.31)	0.745
Exercise self-efficacy ^c^								
Sticking to it	0.49 (−0.19, 1.18)	0.49 (−0.09, 1.08)	0.00 (−0.57, 0.57)	0.997	0.05 (−0.38, 0.48)	0.06 (−0.46, 0.57)	0.00 (−0.57, 0.56)	0.991
Making time for exercise	0.41 (−0.54, 1.37)	0.34 (−0.47, 1.15)	0.08 (−0.71, 0.87)	0.838	0.19 (−0.24, 0.62)	0.00 (−0.51, 0.52)	0.19 (−0.38, 0.75)	0.504
Eating self-efficacy ^c^								
Sticking to it	−0.08 (−0.91, 0.76)	0.38 (−0.33, 1.09)	−0.46 (−1.15, 0.23)	0.179	0.06 (−0.30, 0.42)	−0.16 (−0.57, 0.25)	0.22 (−0.21, 0.66)	0.306
Reducing calories	0.69 (0.03, 1.35)	0.68 (0.12, 1.24)	0.01 (−0.54, 0.56)	0.968	0.25 (−0.15, 0.66)	−0.43 (−0.91, 0.06)	0.68 (0.15, 1.21)	0.013

Data are presented as least squares means with 95% CIs from linear regression models with adjustment for age, education, work, parity, baseline BMI, baseline self-weighing, risk perception of excess GWG, risk perception of GDM, and baseline eating self-efficacy (sticking to it). *p* value for the intervention effect. ^a^ 1 = usually choose low-fat; 3 = rarely or never choose low-fat. ^b^ 1 = not at all confident; 10 = totally confident. ^c^ 1 = not at all confident; 5 = extremely confident.

**Table 3:** Other behavioral measures at 6 weeks postpartum.

Variables	Australian-Born (*n* = 76)			Non-Australian-Born (*n* = 126)		
	Intervention (*n* = 39)	Control (*n* = 37)	*p* Value	Intervention (*n* = 65)	Control (*n* = 61)	*p* Value
Frequent self-weighing	57.7	53.3	0.744	72.3	36.6	<0.001
Perceived change to physical activity	65.4	66.7	0.920	89.4	65.9	0.008
Increased number of regular physical activity sessions	30.8	36.7	0.642	31.1	24.4	0.488
Increased time spent on physical activity sessions	3.9	16.7	0.200 ^a^	6.7	9.8	0.704 ^a^
Increased physical intensity of exercise sessions	0.0	10.0	0.240 ^a^	2.2	9.8	0.188 ^a^
Perceived change to diet	88.5	86.7	1.000 ^a^	97.9	73.2	<0.001
Increased fruit and vegetable consumption	42.3	44.8	0.851	77.8	55.0	0.026
Increased low-fat dairy products	38.5	24.1	0.251	60.0	35.0	0.021
Decreased fruit juice, cordial and soft drink consumption	19.2	27.6	0.467	42.2	37.5	0.657
Decreased intake of snack foods	46.2	41.4	0.722	53.3	30.0	0.030
Decreased takeaway and convenience foods	38.5	51.7	0.324	55.6	20.0	<0.001

Data are presented as percentages. ^a^ Fisher’s exact test due to expected cell frequencies of <5.

## Data Availability

The data presented in the current study are available from the corresponding author on reasonable request.

## Supplementary Materials

The following supporting information can be downloaded at: https://www.mdpi.com/article/10.3390/nu14142988/s1, Figure S1: CONSORT diagram; Table S1: Intervention effect on weight change from baseline to 6 weeks postpartum according to country of birth and baseline BMI; Table S2: Univariate regression analysis for predictors of weight change from baseline to 6 weeks postpartum; Table S3: Multivariable regression analysis for predictors of weight change from baseline to 6 weeks postpartum.
