# Supplementary material for: Limiting Postpartum Weight Retention in Culturally and Linguistically Diverse Women: Secondary Analysis of the HeLP-her Randomized Controlled Trial
Source: Nutrients. 2022 Jul 21;14(14):2988. doi: 10.3390/nu14142988 (PMC9316445; doi:10.3390/nu14142988)
Supplement: Supplementary file 1 [file nutrients-14-02988-s001.zip › nutrients-1784327-supplementary.pdf]

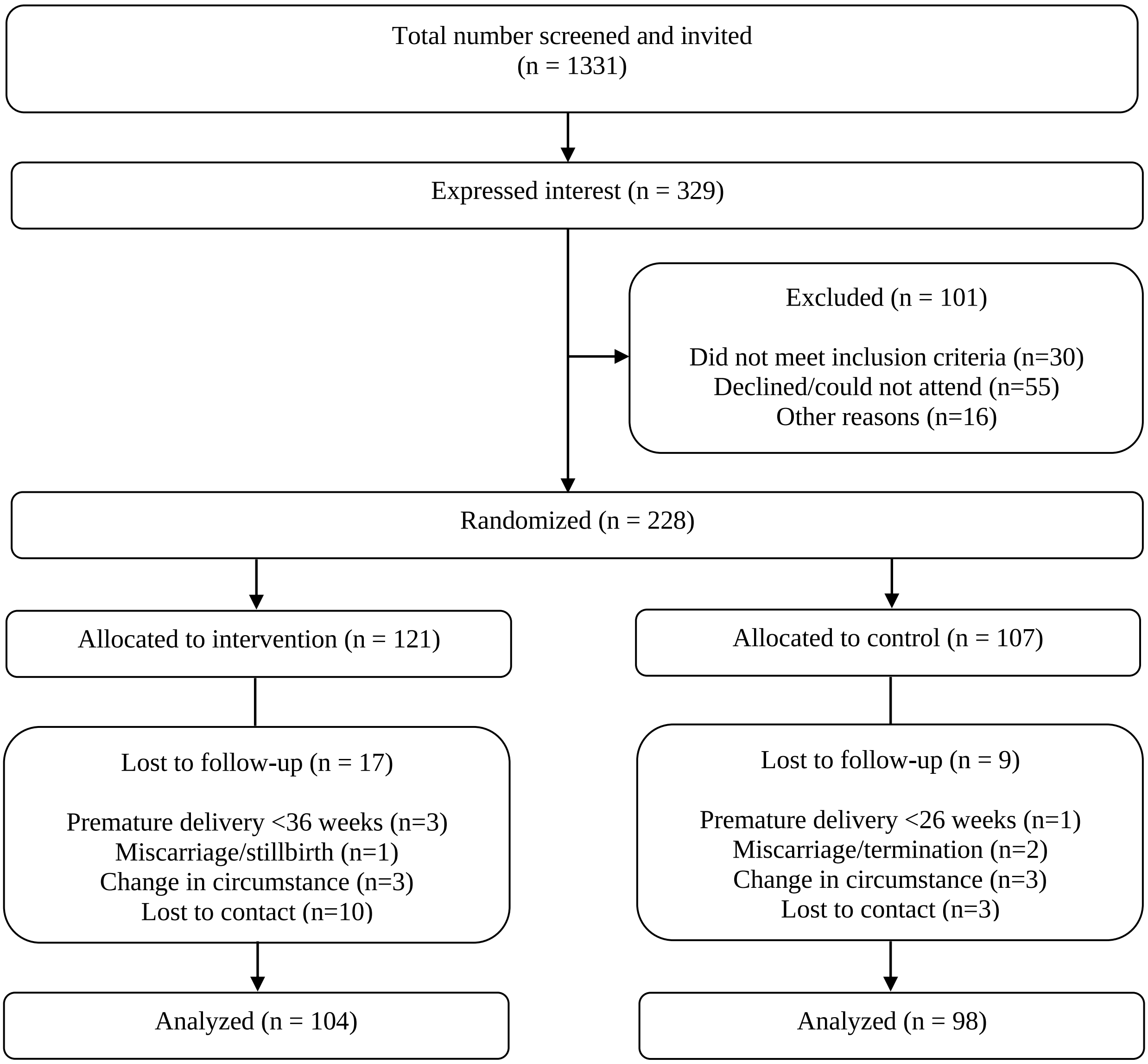

Figure S1. CONSORT diagram

Table S1. Intervention effect on weight change (kg) from baseline to 6 weeks postpartum according to country of birth and baseline BMI

	Intervention		Control		*P* value
	n	Mean±SD	n	Mean±SD	
Australian-born					
Overweight	9	-1.17±2.45	11	0.47±4.93	0.376
Obesity	30	-0.43±5.42	26	-1.26±5.33	0.567
Non-Australian-born					
Overweight	50	1.97±3.92	49	3.98±5.53	0.040
Obesity	15	-1.42±3.77	12	2.03±5.03	0.052

BMI, body mass index; SD, standard deviation.

Table S2. Univariate regression analysis for predictors of weight change (kg) from baseline to 6 weeks postpartum

Variables	Australian-born		Non-Australian-born	
	β (95%CI)	*P* value	β (95%CI)	*P* value
**Group**				
Intervention (vs. control)	0.14 (-2.16, 2.45)	0.901	-2.41 (-4.11, -0.71)	0.006
**Demographics**				
Age (years)	-0.14 (-0.40, 0.13)	0.303	-0.40 (-0.58, -0.22)	<0.001
Education				
Certificate/diploma (vs. high school or below)	2.29 (-0.59, 5.17)	0.118	-2.02 (-5.10, 1.07)	0.198
Bachelor degree or higher (vs. high school or below)	1.39 (-1.77, 4.54)	0.384	0.18 (-2.54, 2.90)	0.895
Work				
Full-time (vs. no paid work)	1.35 (-1.90, 4.61)	0.410	0.98 (-1.13, 3.09)	0.361
Part-time (vs. no paid work)	2.24 (-0.46, 4.95)	0.103	-0.10 (-2.39, 2.18)	0.929
Household income				
$40,000-80,000 (vs. <$40,000)	-0.69 (-4.02, 2.63)	0.677	-0.74 (-2.93, 1.45)	0.505
>$80,000 (vs. <$40,000)	-1.57 (-5.06, 1.92)	0.373	-2.16 (-4.95, 0.62)	0.127
Parity				
Multiparous (vs. primiparous)	1.23 (-1.35, 3.82)	0.345	-3.78 (-5.43, -2.14)	<0.001
Current breastfeeding				
Yes (vs. no)	-1.09 (-4.24, 2.07)	0.493	1.98 (-2.41, 6.37)	0.373
**Anthropometrics**				
Obesity (vs. overweight)	-0.55 (-3.17, 2.06)	0.675	-2.85 (-4.92, -0.78)	0.007
**Behavioral**				
Physical activity change (1000 steps/day)	-0.02 (-0.12, 0.08)	0.723	-0.19 (-0.40, 0.02)	0.081
Fat-related dietary behaviors change [a]	-4.19 (-12.20, 3.81)	0.296	0.49 (-2.87, 3.86)	0.771
Postpartum self-weighing				
Frequent (vs. not frequent)	-0.97 (-3.84, 1.91)	0.503	-1.17 (-3.20, 0.86)	0.255
**Psychosocial**				
Baseline perceived risk of excess GWG (vs. no)	1.38 (-2.62, 5.39)	0.492	-0.66 (-3.03, 1.72)	0.585
Baseline perceived risk of GDM (vs. no)	-1.44 (-4.35, 1.47)	0.327	-0.62 (-2.78, 1.53)	0.566
Weight control confidence change [b]	0.30 (-0.56, 1.17)	0.479	-0.33 (-0.87, 0.21)	0.227
Exercise self-efficacy change [c]				
Sticking to it	0.16 (-1.69, 2.00)	0.865	0.68 (-0.69, 2.05)	0.326
Making time for exercise	0.17 (-1.73, 2.07)	0.859	-0.69 (-2.08, 0.69)	0.323
Eating self-efficacy change [c]				
Sticking to it	0.22 (-2.08, 2.52)	0.846	-1.22 (-2.29, -0.14)	0.027
Reducing calories	-0.37 (-2.67, 1.94)	0.750	-0.78 (-2.07, 0.51)	0.232
**Others**				
Perceived change to physical activity (vs. no)	0.25 (-2.78, 3.29)	0.867	0.11 (-2.36, 2.58)	0.930
Increased number of regular physical activity sessions (vs. no)	-2.10 (-5.08, 0.88)	0.164	-0.24 (-2.55, 2.07)	0.836
Increased time spent on physical activity sessions (vs. no)	-4.97 (-9.41, -0.53)	0.029	1.76 (-2.01, 5.53)	0.357
Increased physical intensity of exercise sessions (vs. no)	-1.02 (-7.40, 5.35)	0.748	-1.23 (-5.65, 3.19)	0.583
Perceived change to diet (vs. no)	2.31 (-1.98, 6.61)	0.286	-3.10 (-5.99, -0.22)	0.035
Increased fruit and vegetable consumption (vs. no)	1.83 (-1.07, 4.74)	0.212	-2.03 (-4.18, 0.12)	0.065
Increased low fat dairy products (vs. no)	-0.43 (-3.59, 2.74)	0.788	-0.28 (-2.34, 1.79)	0.791
Decreased fruit juice, cordial and soft drink consumption (vs. no)	-3.20 (-6.53, 0.12)	0.059	-2.08 (-4.14, -0.02)	0.048
Decreased intake of snack foods (vs. no)	-0.55 (-3.50, 2.39)	0.708	-2.74 (-4.74, -0.73)	0.008
Decreased takeaway and convenience foods (vs. no)	2.03 (-0.85, 4.92)	0.163	-1.39 (-3.49, 0.71)	0.192

CI, confidence interval; GWG, gestational weight gain; GDM, gestational diabetes mellitus.

[a] 1=usually choose low fat; 3=rarely or never choose low fat.
[b] 1=not at all confident; 10=totally confident.
[c] 1=not at all confident; 5=extremely confident.

Table S3. Multivariable regression analysis for predictors of weight change (kg) from baseline to 6 weeks postpartum

Variables	β (95%CI)	*P* value
Australian-born		
Intervention (vs. control)	-0.40 (-3.46, 2.65)	0.792
Age (years)	-0.08 (-0.44, 0.27)	0.643
Multiparous (vs. primiparous)	1.21 (-2.15, 4.58)	0.472
Obesity (vs. overweight)	-0.66 (-4.03, 2.70)	0.693
Increased time spent on physical activity sessions (vs. no)	-5.22 (-10.06, -0.37)	0.035
Non-Australian-born		
Intervention (vs. control)	-1.99 (-3.95, -0.03)	0.047
Age (years)	-0.22 (-0.46, 0.02)	0.076
Multiparous (vs. primiparous)	-1.89 (-3.94, 0.16)	0.070
Obesity (vs. overweight)	-0.43 (-2.77, 1.92)	0.717
Decreased intake of snack foods (vs. no)	-2.21 (-4.25, -0.17)	0.034

CI, confidence interval.
